# End-Cretaceous akaganéite as a mineral marker of Deccan volcanism in the sedimentary record

**DOI:** 10.1038/s41598-017-11954-y

**Published:** 2017-09-13

**Authors:** Eric Font, Julie Carlut, Céline Rémazeilles, Tamsin A. Mather, Anne Nédélec, José Mirão, Sandra Casale

**Affiliations:** 1 IDL-FCUL, Instituto Dom Luís, Faculdade de Ciências da Universidade de Lisboa, Campo Grande, Lisbon, Portugal; 2 Institut de Physique du Globe de Paris, Sorbonne Paris cité, Univ. Paris Diderot, UMR 7154 CNRS Paris cedex 05, France; 3 Laboratoire des Sciences de l’Ingénieur pour l’Environnement, Pôle Sciences et Technologie, Avenue Michel Crépeau, 17042 La Rochelle Cedex 1, France; 4 Department of Earth Sciences, University of Oxford, Oxford, UK; 5 GET- OMP, Université de Toulouse III, Toulouse, France; 6 HERCULES Centre, ECT-Geosciences Department, University of Évora, Évora, Portugal; 7 Sorbonne Universités, Université Pierre et Marie Curie (UPMC), CNRS, UMR 7197, Laboratoire de Réactivité de Surface (LRS), Paris, France

## Abstract

An enigmatic chloride-rich iron (oxyhydr)oxide has been recently identified together with mercury anomalies in End-Cretaceous marine sediments coeval with the Deccan Traps eruptions. The mineral was observed in Bidart (France) and Gubbio (Italy), suggesting a widespread phenomenon. However, the exact nature and origin of this Cl-bearing mineral remained speculative. Here, we characterized the accurate composition and nanostructure of this chloride-rich phase by using micro-Raman spectroscopy, Transmission (TEM) and Scanning (SEM) Electron Microscopy on Focused Ion Beam foils. We also provide new evidence of its occurrence in Zumaia, a reference KPg section from Spain. Results confirm akaganéite (β-FeOOH) as the main phase, with chloride content of 3–5 atomic weight %. Akaganéite particles are constituted by the aggregation of nanorods of akaganéite. Internal structures contain empty spaces, suggesting formation in a low-density (atmospheric) environment. This new mineralogical evidence supports the hypothesis that the observed akaganéite was formed in the Deccan volcanic plume and was transported to the Atlantic and Tethysian realms through the stratosphere. Therefore, akaganéite provides a potential new sedimentary marker to identify the imprint of the Deccan eruptions in the stratigraphic record and is evidence of volcanic halogen degassing and its potential role for the Cretaceous-Tertiary mass extinction.

## Introduction

Emission of sulphur and halogen species from continental flood basalt provinces are of particular interest due to their short-term impact on the environment and climate, and therefore their potential role in the triggering of Phanerozoic mass extinctions^1–3^. Notably, sulfuric acid aerosol (H_2_SO_4_), largely formed by the oxidation of magmatic sulfur dioxide (SO_2_) in the atmosphere, is proposed to have resulted in widespread acid rains and surficial oceanic acidification leading to a major breakdown in the biological productivity and subsequent biotic crises^1^. Another climatic forcing resulting from volcanogenic sulfur degassing is the formation of sulfate aerosols in the lower stratosphere, which would have induced tropospheric cooling due to the reduction of the solar energy flux reaching Earth’s surface^4^. Less attention has been given to halogen species, like hydrochloric acid (HCl), despite their potentially critical effect on ozone destruction and resulting climate change^5^. This is partly because numerical models have suggested that halogen gases from explosive volcanoes are scavenged by aqueous phases before they reach the stratosphere, thus limiting their impact on the climate on a global/regional scale^6^. Although measurements of halogens in the stratosphere after volcanic eruptions remain ambiguous, more recent modelling and *in-situ*/satellite HCl measurements of recent explosive volcanic eruptions, such as the Hekla and Soufrière Hills volcanoes, in 2000 and 2006, respectively, suggest that significant (at Hekla measurements suggest almost all HCl emitted reaches the stratosphere) amounts of HCl can penetrate into the stratosphere^7–10^. In the case of large igneous provinces, these uncertainties are compounded by further debate regarding whether flood basalt eruptions were able to inject significant gas into the stratosphere at all^11, 12^. The recent discovery of Cl-bearing iron (oxyhydr)oxides in marine sedimentary layers from Bidart (Basque Cantabric basin) and Gubbio (Tethys)^13,14^, coeval with the major Deccan eruptions 8000 km distant from these locations during the Cretaceous, could be key evidence for the mode of release and environmental reach of chlorine compounds and other volatiles from Deccan volcanism. If these enigmatic mineral phases can be confirmed as originating from the Deccan volcanism and their origin understood, this would provide compelling evidence for stratospheric injection of the halogen-bearing plume. It would also provide a potential new marker for large-scale volcanism in the stratigraphic record.

**Figure 1:**
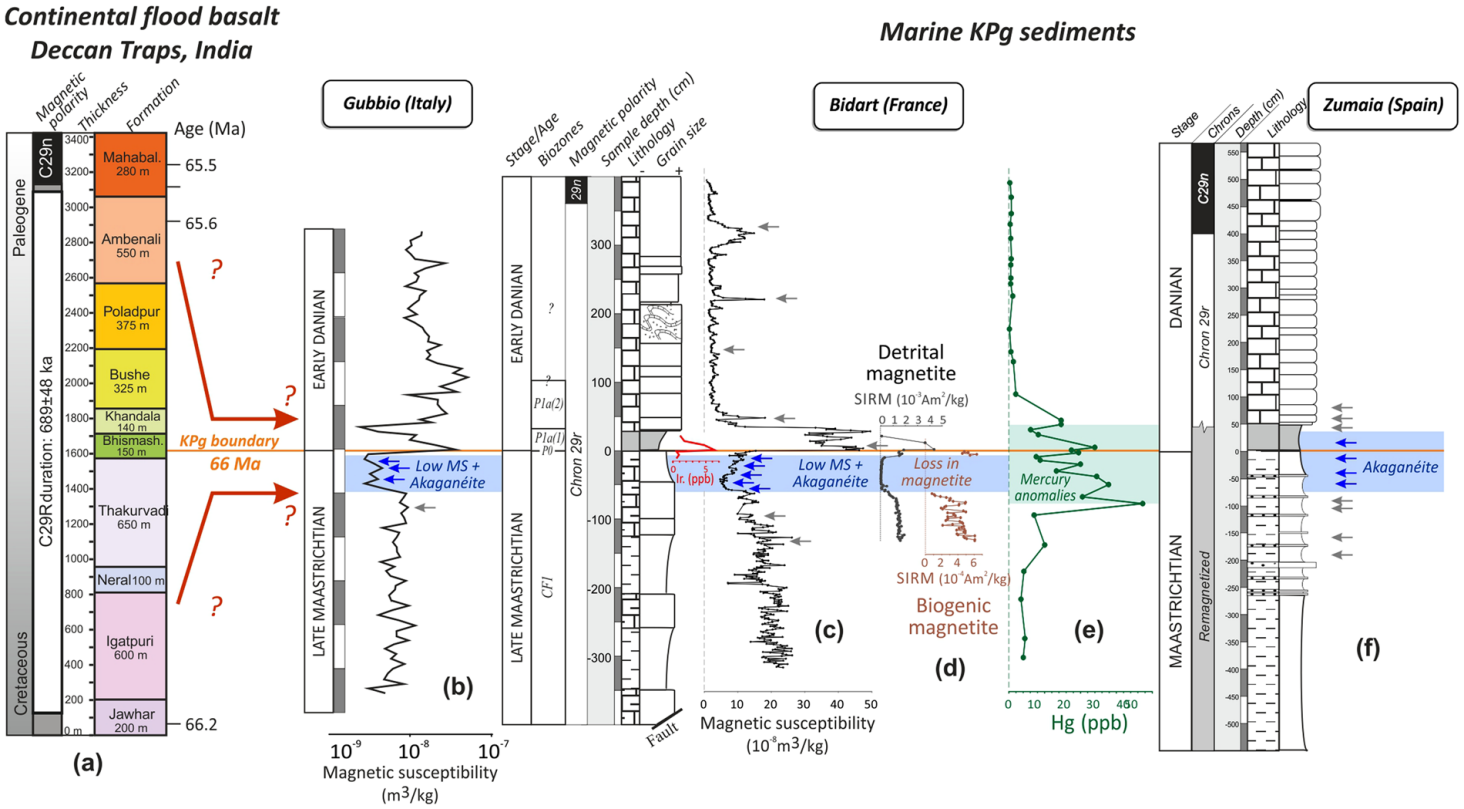
Correlation of (**a**) the age of the Deccan lavas flow in India (U-Pb dating on zircon)^15^ with the KPg marine sedimentary record marked by (**b**) the low MS interval at Gubbio (Italy)^50^; (**c**) the low MS interval containing akaganéite^13^, (**d**) the depletion in detrital and biogenic magnetite^14^, and (**e**) mercury anomalies at Bidart (France)^19^; and (**f**) the interval containing akaganéite at Zumaia (this study). Blue and grey arrows correspond to samples where akaganéite was and was not observed, respectively.

This Cl-bearing iron (oxyhydr)oxide is found in pelagic sediments spanning a ~50 cm thick interval located below the Cretaceous-Paleogene boundary (KPB) at Bidart (France) and Gubbio (Italy), and included within stratigraphic intervals correlated to the Deccan Traps eruptions based on mercury anomalies, paleomagnetic and radiometric data^13–19^. The onset of the most voluminous Deccan episode is estimated to start some 250,000 years before the KPB and end some 500,000 years after the KPB (Fig. 1), with a maximum duration of 750,000 years, based on U-Pb dating of zircon^15^ and high-precision ^40^Ar/^39^Ar dating of plagioclases^16, 17^ from Deccan rocks of the Western Ghats. Within this interval volcanism occurred in limited short megapulse events, each lasting decades to centuries according to paleomagnetic data^18^. The Cl-bearing (oxyhydr)oxide is uniquely observed in stratigraphic intervals characterized by low values of magnetic susceptibility, regarded as the result of enhanced iron oxide dissolution in on-land sediments due to acid rains acting on the continent. The same low magnetic susceptibility interval contains high mercury concentrations^19^, providing further evidence of coeval emission of volcanic gaseous elemental mercury in the atmosphere. Therefore, the stratigraphic position of this mineral and its association with evidence of environmental perturbation suggest an origin related to volcanic activity. A previous study speculated, based on Scanning Electron Microscopy (SEM) observations coupled to Energy Dispersive (EDS) analyses from the Bidart samples, that this mineral was akaganéite^14^, a rare mineral on Earth but common on Mars^20^. However, SEM-EDS techniques on their own provide only semi-quantitative compositional data and, it has not been possible to date to identify akaganéite by using common classical bulk mineralogical techniques (XRD, XRF, Mossbauer, etc.) due to its very low concentration in the sediment, below the detection limit of most techniques.

In this study, we conduct micro-Raman spectroscopy and high-resolution Scanning (SEM) and Transmission Electron Microscope (TEM) analysis on single Cl-rich grains previously localized in rock fragments from Bidart and subsequently extracted by using Focus Ion Beam (FIB) techniques, in order to better constrain the composition and microstructure of the Cl-bearing iron (oxyhydr)oxide and discuss its possible formation mode and transportation to the marine sediments. The Cl-bearing grains observed in the Gubbio samples^13^ were too small (<5 μm) to be analysed by using micro-Raman spectroscopy. Instead, we analysed samples from the Zumaia section, another well-constrained KPg section of the Basque Cantabric basin^21^ and located some 70 km from Bidart. Our findings provide new insights into the nature of this mineral and its origin with implications in terms of stratigraphic proxies for large-scale volcanism and the global environmental impacts of LIPs.

## Results

### Scanning Electron Microscopy (SEM)

**Figure 2:**
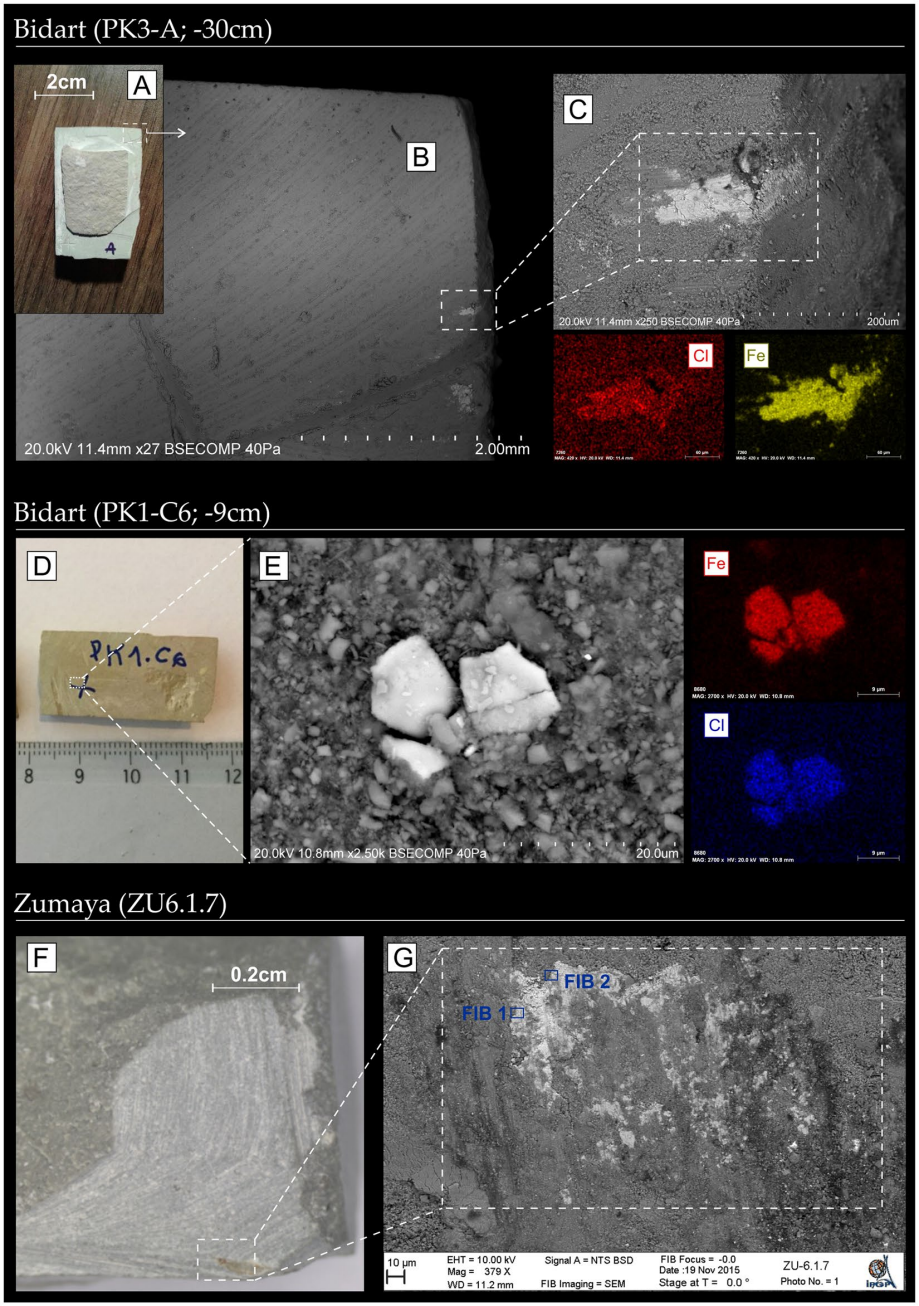
Optical photographs of the rock fragments under study showing the location of akaganéite grains further observed under SEM. (**A**) Photograph of the PK3-A (Bidart) rock fragment and (**B**,**C**) SEM back-scattered image of the studied akaganéite together with compositional maps (Fe and Cl); (**D**) Photograph of the PK1-C6 (Bidart) rock fragment and (**E**) SEM back-scattered image and compositional maps of akaganéite. (**F**) Photograph of the ZU6.1.7 (Zumaia) rock fragment and (**G**) SEM back-scattered image of akaganéite. Position of FIB 1 and 2 extracted from sample ZU6.1.7 are shown (see Figs 3 and 5). Distance in cm refers to sample depth from the KPg boundary.

In Bidart and Gubbio, Cl-grains are only observed in the low magnetic susceptibility interval. In Zumaia, Cl-bearing grains are observed below and just above the KPB (Fig. 1). In all studied sections, we also carefully analysed samples located below this low magnetic susceptibility interval and samples located above the KPB, in the Danian carbonates. After analysing more than 150 iron oxides based on EDS spectra, no akaganéite was found. The interval where akaganéite is observed is illustrated in Fig. 1. Most Cl-bearing grains observed under SEM have a plate-like shape well-embedded in concordance with the deposition plane of the sediment (Fig. 2), thus suggesting a primary depositional origin. Further evidence of their primary origin is the absence of crystallisation in fractures, voids or upon host minerals and the sparse distribution of the grains. Grain-sizes vary from 20 to 100 μm (Fig. 2). Grains have platy or flake-like morphologies, with no evidence of erosion or abrasion, suggesting a proximal detrital source or aeolian transport (Supplementary Information).

**Figure 3:**
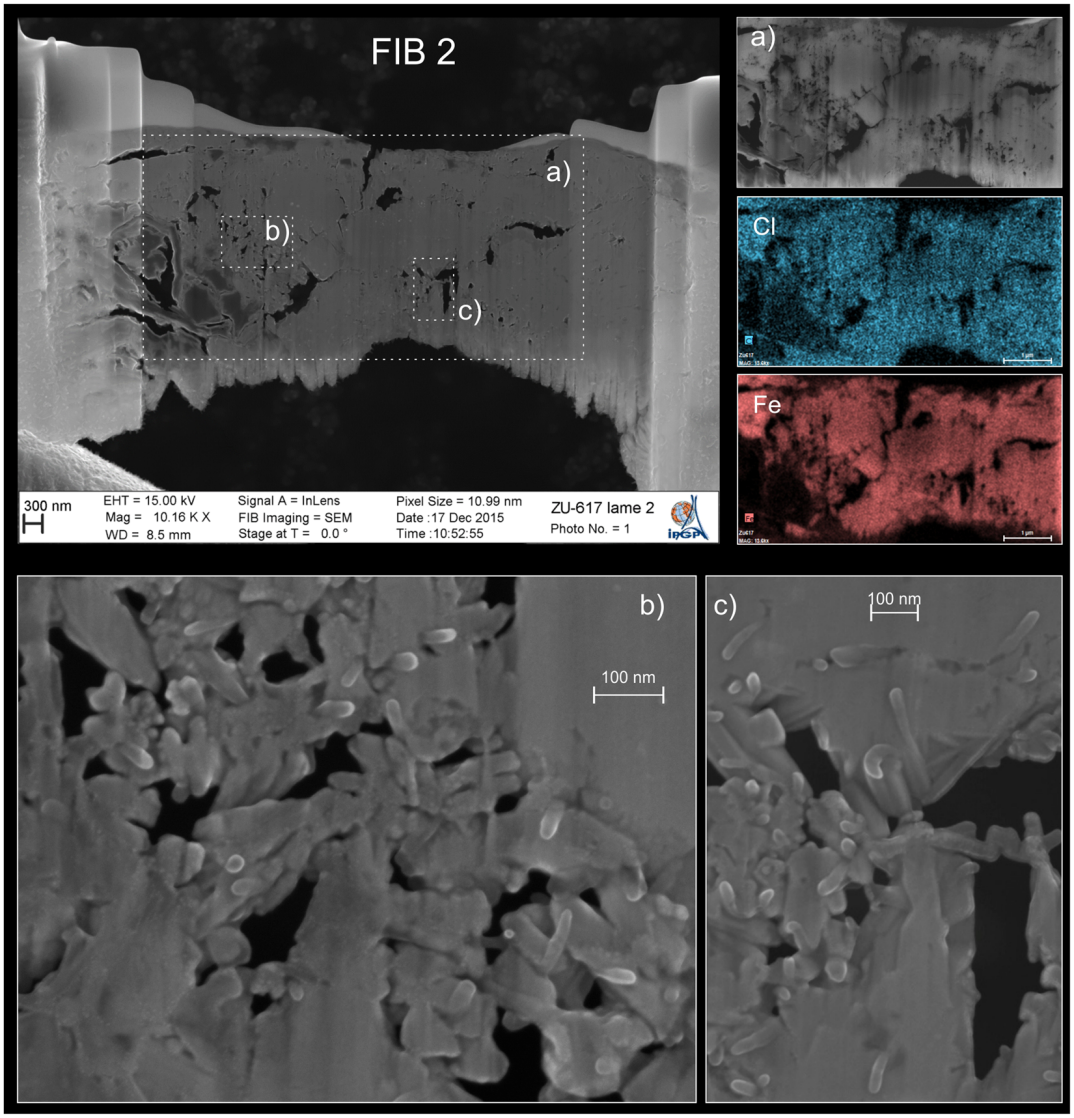
TEM observation of FIB foil 2 (sample ZU6.1.7). Insets (**a**) show concentric and porous features, with the presence of Cl and Fe as main constituting elements. (**b**,**c**) High-resolution images of the microstructure reveal that the grain is constituted by the aggregation of individual somatoidal nanoparticles of akaganéite.

High-resolution SEM images of FIB 2 foil are shown in Fig. 3. Element Semi-Quantitative Analyses made on two 800 × 800 nm regions give average values of Fe at 88 at. % and Cl at 12 at. % (excluding O), leading to Fe/Cl atomic ratio of 7.3. This value is fully compatible with estimation of the 1–7% chloride content of akaganéite described by^22^. Electron Dispersive X-ray Spectroscopy mapping, performed on a large area of the section, reveals no significant variations in composition with a range of Fe/Cl ratio between 7.0 and 8.6 (Fig. 3c). The observation of FIB 2 foil also reveals a porous texture with concentric growth features and numerous void spaces (Fig. 3). Observation at a finer scale shows that the so-called grains are constituted by the agglomeration of individual and somatoidal-like structures of 100–200 nm in length and 25–30 nm in diameter, typical of akaganéite nanorods described in the literature^23–25^.

### Micro-Raman spectrometry

**Figure 4:**
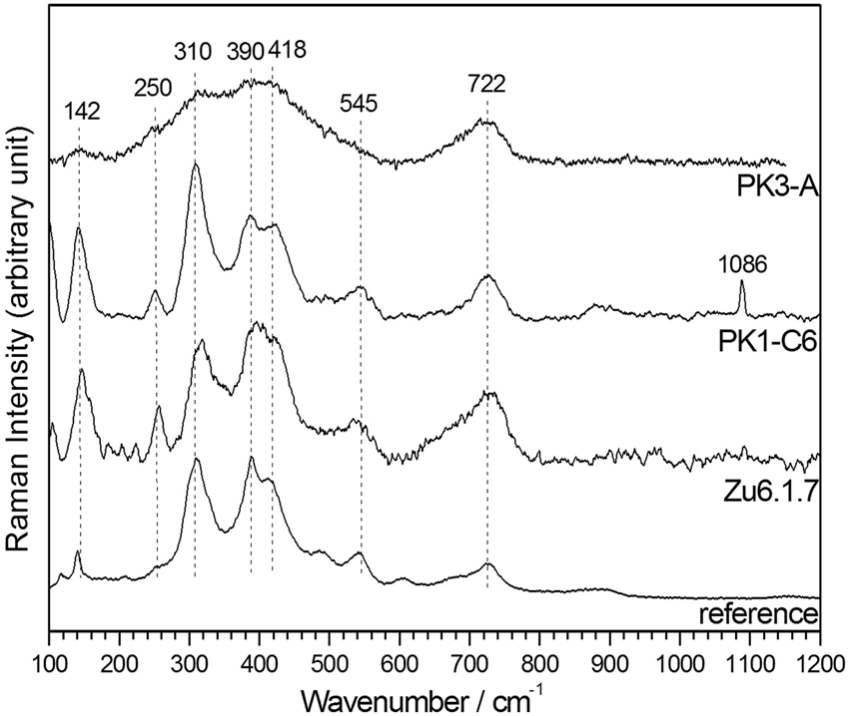
Micro-Raman spectra of akaganéite grains observed in samples from Bidart (PK3-A and PK1-C6) and Zumaia (Zu.6.1.7). Reference spectra of akaganéite is provided for comparison^26^.

Figure 4 displays spectra obtained from several grains localized on samples PK3-A, PK1-C6 (Bidart) and ZU6.1.7 (Zumaia). A reference of an akaganéite powder synthesized by precipitation and oxidation of a mixture of FeCl_2_, 4H_2_O and NaOH solutions (R′ = 8)^26^ is shown for comparison. The spectra obtained from the rock samples correspond unambiguously to akaganéite with characteristic bands at 310, 390, 418, 545 and 722 cm^−1^. The spectra were corrected for the fluorescence signal due to the silicate matrix. The akaganéite grain analyzed on sample PK3-A seemed to have been crossed by the cutting instrument during the sampling. A light heating resulting from this could have affected its crystallinity, leading to broad bands on the spectrum. Additional bands are also observed. The band at 250 cm^−1^ is particularly intense for samples PK1-C6 and ZU6.1.7. This band is present in the akaganéite spectrum, in general with a very low intensity, and likely corresponds to lepidocrocite (δ-FeOOH). The band at 142 cm^−1^ for samples PK1-C6 and ZU6.1.7 likely corresponds to FeCl_3_. Finally, the peak at 1086 cm^−1^ observed on the spectrum of sample PK1-C6 is characteristic of the ν1 symmetric stretching mode of CO_3_^2−^ and can be attributed to calcite (CaCO_3_).

### Transmission Electron Microscopy (TEM)

**Figure 5:**
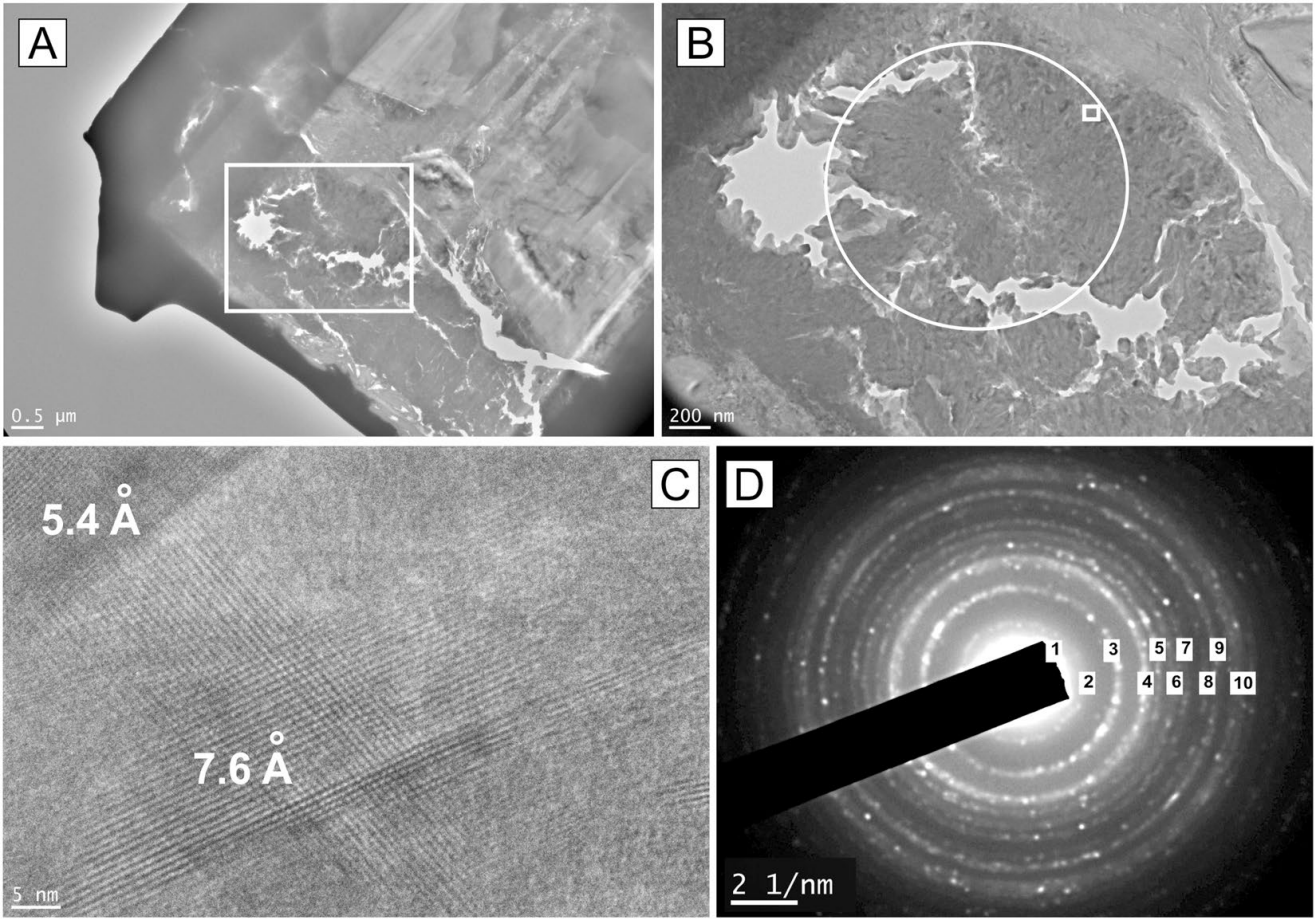
Bright field TEM micrograph from the FIB-1 section extracted from the Zumaia sample ZU.6.1.7. (**A**) General view of the section. (**B**) Magnified area of the white rectangle in a showing the elongated structure of akaganéite grains. (**C**) High resolution image corresponding to small rectangle in b, spacing of the atomic planes was calculated using a profile plot. (**D**) Grainline selected area diffraction (SAED) pattern correspond to the broad area within the circle in b. All the numbered rings have been indexed and found compatible with akaganéite (see text for explanation).

The micro-/nanostructure was further examined with high-resolution transmission electron microscopy (HRTEM) on FIB 1 foil (Fig. 5A). The images reveal that the sample contains multiple voids and somatoidal structures about 50–70 nm large (Fig. 5B), as already observed on FIB 2 foil using the SEM. The various growth directions are clearly evidenced by the high-resolution images showing the atomic planes (Fig. 5C). The lattice spacing obtained by selected area electron diffraction (SAED) on a 1.4 μm diameter area (represented by a circle in Fig. 5B), shows a powder like pattern (Fig. 5D). The first ten d-spacings were determined and they all correspond, within an uncertainty of ~0.2 Å, with the reference spacing of akaganéite determined after X-ray diffraction data (RRUFF database) (Table 1).

**Table 1:** (1) First 10 most intense Akaganéite X-ray peak intervals (Intensity I/IMax above 0.1) – Compiled from RRUFF database after^39, 48, 49^.

D-space Akaganeite^(1)^	D-space from SAED^(2)^
7.46 Å	7.3 Å (1)
5.26–5.29 Å	5.5 Å (2)
3.32–3.35 Å	3.4 Å (3)
2.63–2.65 Å	2.6 Å (4)
2.55 Å	2.4 Å (5)
2.29–2.30 Å	2.2 Å (6)
2.10 Å	2.0 Å (7)
1.95–1.96 Å	1.8 Å (8)
1.75–1.76 Å	1.7 Å (9)
1.64–1.65 Å	1.6 Å (10)

(2) Measurements from the SAED pattern (this study) indexed using the Gatan software “Digital Micrograph”. Numbers in parenthesis are referenced from Fig. 5D.

## Discussion

Here, we present clear evidence that the enigmatic Cl-containing oxyhydroxide observed in marine (pelagic) Cretaceous sediments from Bidart, Zumaia, and Gubbio corresponds to akaganéite (β-FeOOH) (Fig. 2 and supplementary information). Akaganéite is a geologically rare mineral that was first described as a weathering product of pyrrhotite in the Japanese limonite mine Akagané^27^. Akaganéite is the β phase of the ferric (oxyhydro)oxide FeOOH. It is isostructural with hollandite (BaMnO_2_) and contains chloride ions in tunnels parallel to the c-axis, which are necessary to maintain the structure. It can be easily synthesized in laboratory experiments by hydrolysis of acidic FeCl_3_ solutions or by oxidative hydrolysis of FeCl_2_ solutions^22, 26^. However, it is rare in natural settings due to the particular environmental conditions required for its precipitation, including hyper-chlorinated, acidic and oxidizing conditions, as well as large concentrations of both dissolved ferrous iron and chloride ions^26^. The detection of akaganéite was recently reported on Mars based on remote observations from the CRISM (Compact Reconnaissance Imaging Spectrometer for Mars)^20^. Although the origin and formation mode of the Martian akaganéite remain to be solved, the authors suggest that akaganéite precipitation was favoured by the highly acidic and hyper-chlorinated conditions of the Martian atmosphere^20^.

These environmental conditions are rare on Earth. In our case study on marine sediments, primary precipitation of akaganéite in ocean seawater is thermodynamically unlikely, due to the high solubility of chloride in liquid water, moderately oxidizing conditions, lack of dissolved ferrous iron and the neutral pH of seawater. This suggests that this akaganéite likely formed elsewhere and was transported to the basin by run-off or aeolian processes. The sparse distribution and well-embedded aspect of the grains observed under SEM within the sedimentary matrix support a detrital inherited origin (Fig. 2). The relatively well-preserved shape of the akaganéite grains compared to the severely altered aspect of the detrital iron oxides (magnetite, ilmenite) contained in the same samples argue for aeolian transportation^14^. On Earth, akaganéite has been described in a limited number of natural settings: (i) as a corrosion product of steel in chloride containing environments or chloride-bearing excavated archaeological iron^28^; (ii) as a corrosion product of meteorites^29^; (iii) together with jarosite in lesser amounts, as a natural precipitate from oxidation of iron sulphide minerals during the occasional drying of acidic (pH ~ 2) and chlorinated wetlands, such as the Bottle Bend Lagoon in Australia^30^ - note that saline and acidic Lake Orr and Lake Whurr from Western Australia only contain jarosite, likely because of insufficient salinity (hence insufficient Cl content)^31^; (iv) as a precipitate associated with volcanic hydrothermal fluids or fumaroles^32–34^ on land; and (v) as a rare mineral in sulfide mounds and chimneys related to oceanic ridge hydrothermalism^35^.

Cases (i) and (ii) are ruled out as a source in our Cretaceous sediments. Steel was clearly absent in the Cretaceous. In the case of akaganéite found in meteorites, it has been assigned as the corrosion product of Fe-Ni alloys (kamacite, taenite) contained in a few iron meteorites^29, 36–40^. A detailed study of a suite of 12 iron meteorites from a variety of Antarctic environments showed that akaganéite forms *in situ* by replacement of metal and contains 3–5 wt% Ni (maximum values 19 wt% Ni), reflecting the composition of its parent kamacite^29^. Backscattered-electron images of the Antarctic chondrites show that akaganéite occurs preferentially in veins and fissures, as a secondary mineral replacing kamacite^29^. Further evidence of the presence of Ni-containing akaganéite in meteorites was presented from other regions and environments, such as the Campo del Cielo meteorites in Argentina^40^, or the Barbianello meteorite in Italy^41^. The absence of Ni in the composition of our akaganéite as confirmed by SEM-EDS and micro-Raman analyses (Fig. 4), definitively excludes an extra-terrestrial origin.

Similar conditions to those proposed for Martian akaganéite formation are required for case (iii), where akaganéite precipitation in sulfidic dried wetland is favoured by the extremely acidic and saline water conditions of such an environment. In the Bottle Bend Lagoon in Australia, akaganéite occurs as typical spindle-shaped akaganéite nanoparticles bounded on halite and gypsum grains^30^. In our case, formation of akaganéite from the oxidation of iron sulphides in surroundings dried wetlands and further transportation and deposition into the Basque-Cantabric basin cannot be ruled out, although preservation of such delicate and fragile aggregates during transportation from the continent to the ocean is unlikely. Besides, we also propose that sediments at Gubbio, located in the Tethys during the Cretaceous, contain akaganéite (inferred based on the similarity of the Cl-bearing minerals to the Bidart material^13^). The probability of having such a similar and peculiar environmental source (i.e. sulfidic dried lakes) in two distal sections is rather low. An oceanic hydrothermal origin from the mid-ocean Atlantic ridge (possibility (v) in the list above) also seems unlikely. The Atlantic Ocean (basin containing the Bidart and Zumaia sections) and the Tethys (basin containing the Gubbio section) are poorly connected during the Cretaceous, so the fact that these akaganéite grains are found in the same particular stratigraphic level from these sections in different basins does not support an oceanic ridge hydrothermalism source.

This leaves a terrestrial volcanic origin (iv) as the most likely explanation for the akaganéite documented here. For instance, akaganéite co-occurring with jarosite was described as a surface deposit on andesite boulders near the steaming fumarole vents of the White Island volcano in New Zealand^34^. The fact that we have identified akaganéite in marine sediments from two sections in the Atlantic Ocean (Bidart and Zumaia) and that it is further inferred in a separate and remote basin, i.e. the Tethys realm (Gubbio)^13^, separated by more than 1,500 km, makes a local volcanic source a less likely explanation than a more distant common source associated with a dispersion mechanism. In all the studied sections (Bidart, Zumaia and Gubbio), akaganéite occurrence is restricted to the same stratigraphic levels, more precisely to the last 50 cm below the Cretaceous-Paleogene boundary where magnetic susceptibility gradually decreases to very low values^13^. This restricted stratigraphic position makes volcanic sources from a local source like the Tethys realm, e.g., the Pontide volcanic arc of Turkey, less likely. The Pontide orogenic belt preserves a record of various magmatic events from Jurassic rifting to Neogene post-subduction volcanism^42^. The most active volcanic production was related to subduction of the Tethys Ocean, for which an age of 86–75 Ma has been recently provided by ^206^Pb–^238^U age analyses of zircon and ^40^Ar/^39^Ar age data of plagioclase from the Pontides volcanics^43^. In contrast, ^40^Ar/^39^Ar dating suggested that the most voluminous Deccan eruptions are represented by the Poladpur and Ambenali Formations^16^, and placed the KPg boundary at the base of the Poladpur Formation. Although U-Pb ages placed the KPg boundary between the Poladpur and Ambenali Formations^15^, the broad correlation between the age of the massive Poladpur and Ambenali Formations and the age of the stratigraphic interval where we find akaganéite supports a causal relationship. Further evidence is that the stratigraphic interval containing akaganéite very well correlates with low magnetic susceptibility values and mercury enhancements (Fig. 1). The decrease in magnetic susceptibility results from the dissolution of detrital magnetite and the disappearance of biogenic magnetite, probably due to acid rains triggered by Deccan volcanism and to global environmental acidification^14, 44^. Also documented is the close relationship with mercury anomalies in the same stratigraphic interval at Bidart^19^, which strongly suggest a link with the major eruptions from the Deccan Traps in India (Fig. 1). Therefore, we suggest that all these independent markers (mercury anomalies, low magnetic susceptibility and akaganéite) are the result of the relatively short-lived and most intense Deccan Traps eruptions. Because our studied sections were separated by more than 8000 km from the Deccan Traps during the Cretaceous, and located in the opposite hemisphere (Fig. 1), our results suggest that aerosols released by the Deccan volcanic plume were transported to the North hemisphere through the stratosphere, a well-documented process for other volcanic products following intense volcanic eruptions (e.g., Pinatubo, 1991^45^). The grain size of the observed akaganéite aggregates (10–100 μm) are in the range of ash and tephra documented to have been transported several thousands of kilometres away from volcanic sources^46, 47^. This provides new evidence in support of significant stratospheric injection of material from LIP volcanism^12^, which is an important consideration when modelling the climate and environmental effects of Large Igneous Provinces and their contribution to Phanerozoic mass extinctions.

## Methods

Samples correspond to Upper Cretaceous marine sediments, more exactly to marls in Bidart and Zumaia and carbonates in Gubbio (see Font *et al*.^14^ for a detailed description of the mineralogy of the Bidart samples). Rock fragments, collected from the appropriate stratigraphic levels of the Bidart, Zumaia and Gubbio sections, were collected in the field and cut into small rectangular (~3 × 5 cm long) booklets in the laboratory (Fig. 2A). Samples were then cut into pieces along the equatorial plane (Fig. 2A), which roughly corresponds to the depositional plane (see Fig. 1f in ref. 14). Fresh sample fractures were obtained by cutting the border of the booklet using a diamond saw (Dremel) and then broken in two pieces, avoiding any contact of the saw with the fresh fracture (Fig. 2A). Note that observation from polished thin sections was unsuccessful, because akaganéite grains became detached from the matrix during the abrasion and polishing processes.

We first conducted SEM-EDS analysis on these rock fragments in order to i) localize the Cl-rich grains in the sediment matrix, and ii) to check for the grain size of the Cl-rich grains for subsequent micro-Raman analysis. This technique requires a minimum grain (spot) size of ~10 μm in order to provide an accurate measurement of the composition of the analysed grain. The examination of rock fragments with the SEM is not adequate for accurate compositional EDS analysis (compared to polished thin-sections), but at least provides a three-dimensional picture allowing a rapid and better identification of the shape and morphology of the grains, as well as their relations with the sediment matrix. This is particularly true for iron oxides and hydroxides as they are heavy minerals and easily observable in back-scattered imaging. SEM investigations were conducted using a Hitachi S-3700N SEM microscope coupled to a Bruker XFlash® 5010 EDS detector at the Hercules laboratory (Évora, Portugal). The electron source for the SEM is a tungsten wire. The accelerating voltage is 20 keV. Qualitative compositional analysis was provided by energy dispersive spectra (EDS) by using the ESPRIT Software (Bruker).

Micro-Raman spectroscopy experiments were conducted at the LaSIE laboratory of the University of La Rochelle (France) using a Jobin Yvon High Resolution Raman spectrometer (LabRAM HR) equipped with a microscope (Olympus BX 41) and a Peltier-based cooled charge coupled device (CCD) detector. Measurements were carried out on grains previously analysed by SEM and identified as iron/chloride-containing compounds. Spectra were recorded with the acquisition LabSpec software at room temperature with a resolution of approximately 2 cm^−1^. Excitation was provided by a He-Ne laser at 632.8 nm. The spot under the ×50 lens had a diameter of ~3 μm. The acquisition time was equal to 60 seconds and the laser power was reduced to 0.9 mW in order to prevent the transformation of the analyzed grains into hematite α-Fe_2_O_3_ that can take place due to an excessive heating. In case of fluorescence, the sample was pre-illuminated by the laser beam, until the fluorescence signal decreased and the peaks of the desired compounds appeared clearly. Presented spectra are baseline-corrected (LabSpec software).

Two Focused Ion Beam sections (FIB 1 and FIB 2) were prepared on one of the grains identified by micro-Raman spectroscopy (ZU6.1.7). The areas were covered with platinum prior to FIB milling in order to preserve the surface of the sample. Sections were then milled inside a SEM Zeiss Sigma and were fixed on a copper grid. FIB 2 was carbon coated and used for observations and quantitative compositional analysis inside the same SEM (in In Lens and Back Scattered mode). The interaction volume of the SEM electron beam is minimized thanks to the low thickness (80 nm) of the foil and allows us to perform ultra high-resolution observations and analyses. FIB 1 was used for TEM Bright field High-Resolution Transmission Electron Microscopy (HR-TEM) and EDS analysis performed at the ≪ Institut des Matériaux de Paris Centre ≫ using a JEOL 2010 microscope operating at 200 kV with a LaB6 filament and equipped with an Orius CCD camera (Gatan) and a PGT detector. The measurements from the HR-images and diffraction diagram (Selected Area Electron Diffraction and HRTEM) were performed using the Gatan software “Digital Micrograph”.

### Data availability

The datasets generated during and/or analysed during the current study are available from the corresponding author on reasonable request.

## Electronic supplementary material

Supplementary information
