# Supplementary material for: End-Cretaceous akaganéite as a mineral marker of Deccan volcanism in the sedimentary record
Source: Sci Rep. 2017 Sep 13;7:11453. doi: 10.1038/s41598-017-11954-y (PMC5597636; doi:10.1038/s41598-017-11954-y)

End-Cretaceous akaganéite as a mineral marker of Deccan volcanism in the sedimentary record

E. Font, J. Carlut, C. Rémazeilles, T. A. Mather, A. Nédélec, J. Miraõ and S. Casale.

Bidart (France)

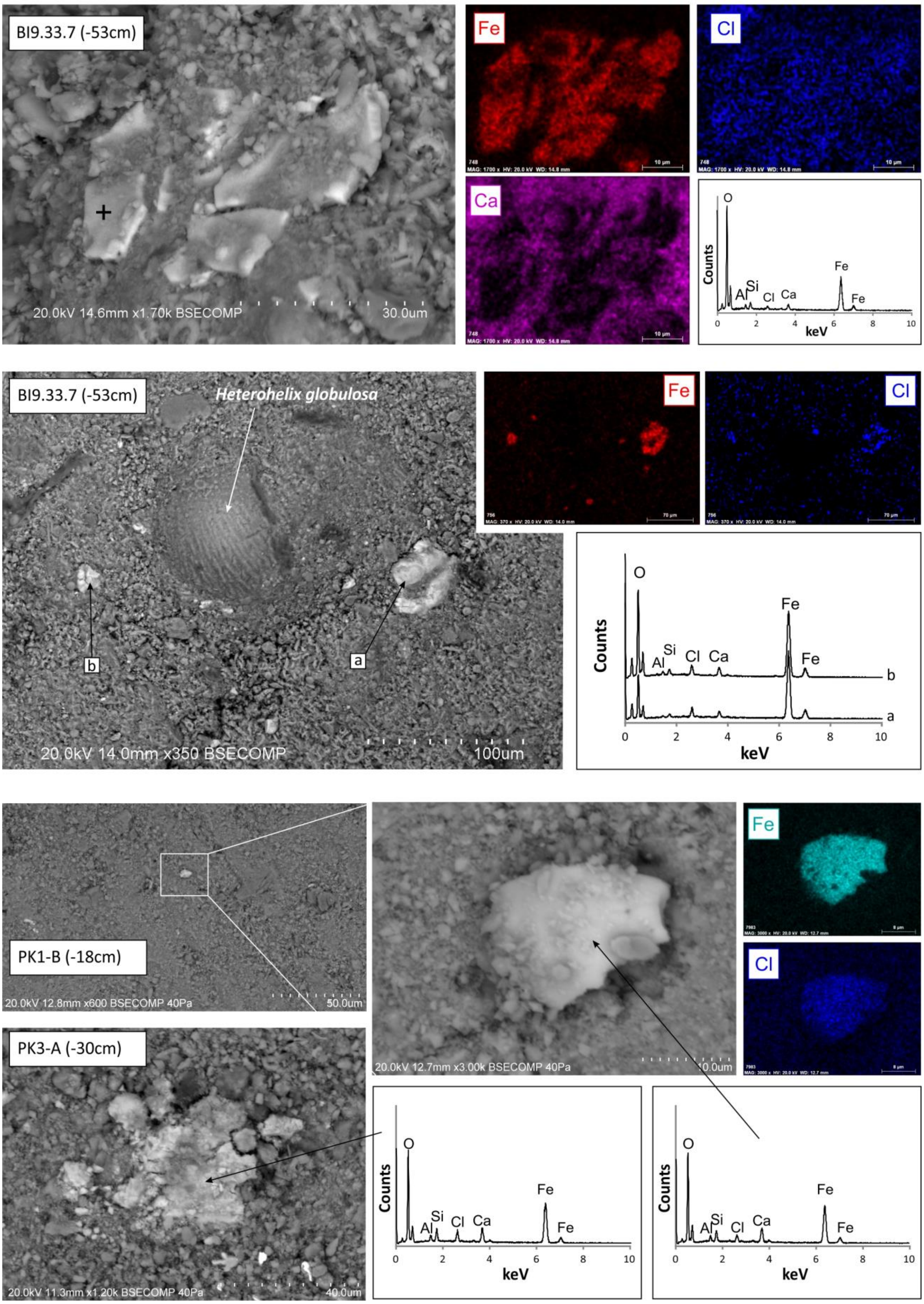

Gubbio (Italy)

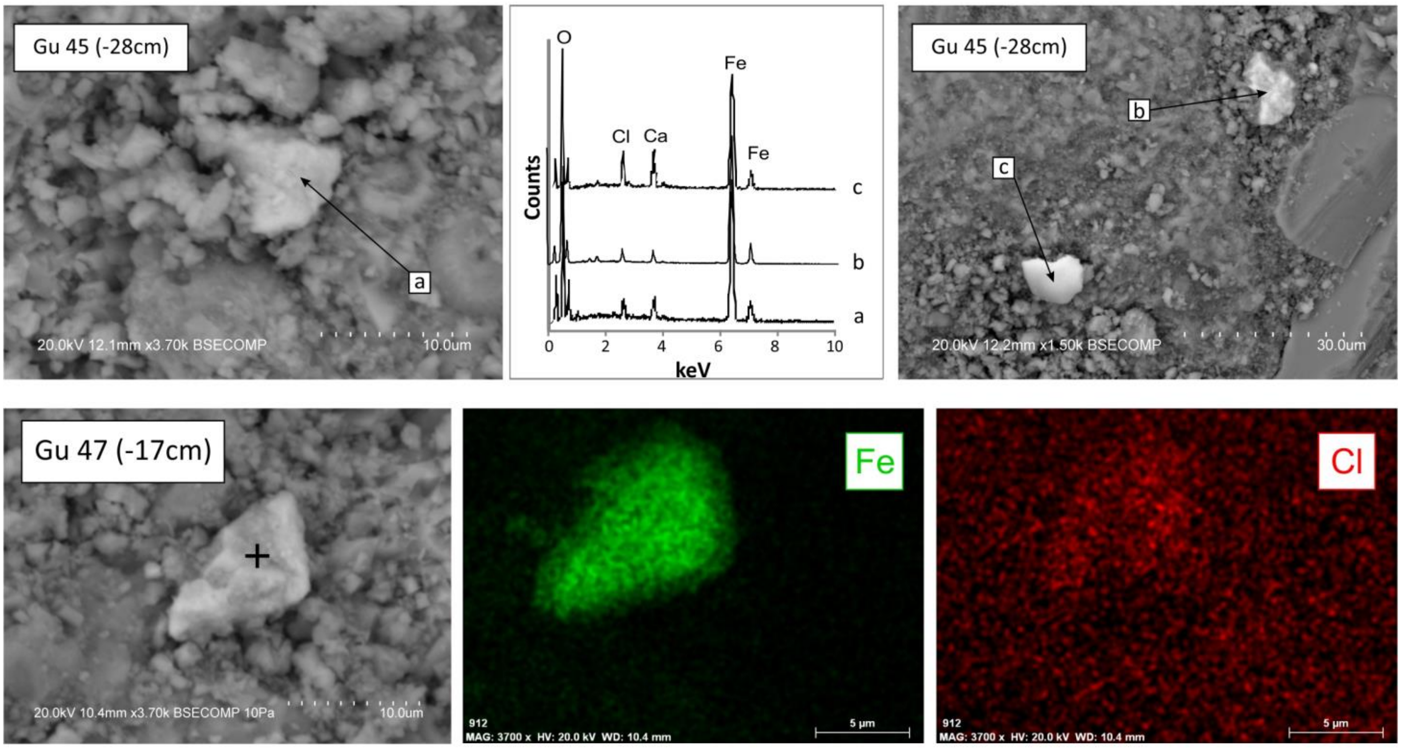

Zumaia (Spain)

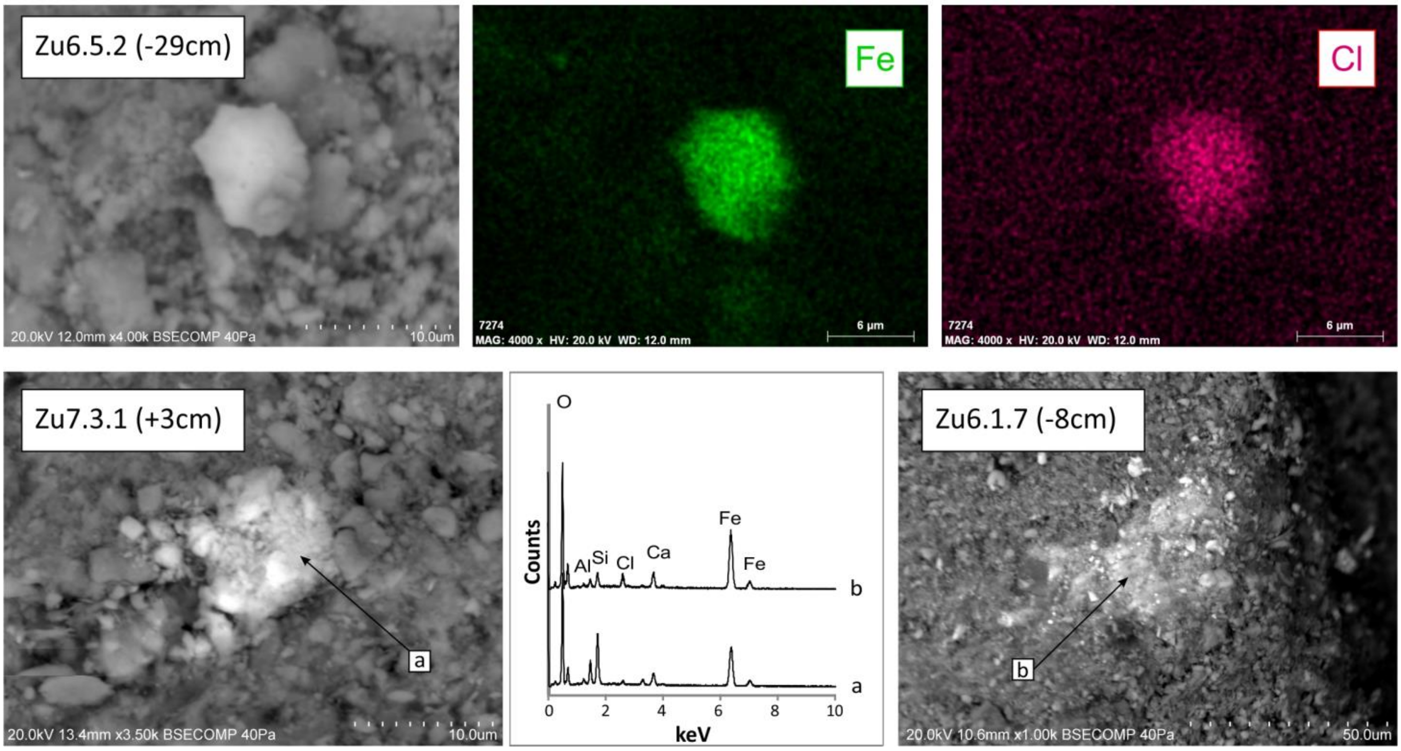

Supplement: Supplementary file 1 — Supplementary information [file 41598_2017_11954_MOESM1_ESM.pdf]
